# Determining the relationship of p16^INK4a^ and additional molecular markers of aging with clinical frailty in hematologic malignancy

**DOI:** 10.1007/s11764-024-01591-6

**Published:** 2024-04-28

**Authors:** Ashley E. Rosko, Mohamed I. Elsaid, Jennifer Woyach, Nowshin Islam, Noah Lepola, Jazmin Urrutia, Lisa M. Christian, Carolyn Presley, Alice Mims, Christin E. Burd

**Affiliations:** 1 Division of Hematology, The Ohio State University, Columbus, OH USA; 2 Department of Biomedical Informatics, The Ohio State University, Columbus, OH USA; 3 Departments of Molecular Genetics, Cancer Biology and Genetics, The Ohio State University, Columbus, OH USA; 4 Department of Psychiatry and Behavioral Health, Institute for Behavioral Medicine Research, The Ohio State University, Columbus, OH USA; 5 Division of Medical Oncology, The Ohio State University, Columbus, OH USA; 6 James Comprehensive Cancer Center, 300 West 10th Ave, Columbus, Ohio 43210 United States

**Keywords:** Hematologic Malignancy, Frailty, Aging biomarkers, p16

## Abstract

**Purpose:**

Older adults with hematologic malignancies (HM) have unique challenges due to age and fitness. The primary aim of this pilot study was to benchmark the ability of multiple biomarkers of aging (p16, epigenetic clocks, T cell gene expression profiles, and T cell receptor excision circles (TREC) to identify frailty as measured by a clinical impairment index (I^2^) in patients with HM.

**Methods:**

70 patients newly diagnosed with HM had peripheral blood T lymphocytes (PBTL) analyzed for p16^INK4a^ expression using the OSU_Senescence Nanostring CodeSet. PBTL epigenetic age was measured using 7 epigenetic clocks, and TREC were quantified by qRT-PCR. A composite clinical impairment index (I^2^) was generated by combining values from 11 geriatric metrics (Independent Activities of Daily Living (iADL), physical health score, Short Physical Performance Battery (SPPB), Body Mass Index (BMI), Eastern Cooperative Oncology Group (ECOG) performance status, self-reported KPS, Blessed Orientation Memory Concentration (BOMC), polypharmacy, Mental Health Inventory (MHI)-17, Medical Outcomes Study (MOS) subscales). Clinical frailty was defined as a score of 7 or greater on the I^2^.

**Results:**

Age-adjusted p16^INK4a^ was similar in newly diagnosed patients and healthy controls (*p* > 0.1). PBTL p16^INK4a^ levels correlated positively with the Hannum [r = 0.35, 95% CI (0.09–0.75); p adj. = 0.04] and PhenoAge [r = 0.37, 95% CI (0.11–0.59); p adj. = 0.04] epigenetic clocks. The discrimination ability of the I^2^ model was calculated using the area under the receiver operating characteristic curve (AUC). After adjusting for chronologic age and disease group, baseline p16^INK4a^ [AUC = 0.76, 95% CI (0.56–0.98); *p* = 0.01], Hannum [AUC = 0.70, 95% CI (0.54–0.85); *p* = 0.01], PhenoAge [AUC = 0.71, 95% CI (0.55–0.86); *p* = 0.01], and DunedinPACE [AUC = 0.73, 95% CI (0.57–0.88); *p* =  < 0.01] measures showed the greatest potential to identify clinical frailty using the I^2^.

**Conclusions:**

Our pilot data suggest that multiple blood-based aging biomarkers have potential to identify frailty in older adults with HM.

**Implications for Cancer Survivors:**

We developed the I^2^ index to quantify impairments across geriatric domains and discovered that PBTL p16, Hannum, PhenoAge, and DunedinPACE are promising indicators of frailty in HM.

**Supplementary Information:**

The online version contains supplementary material available at 10.1007/s11764-024-01591-6.

## Introduction

Older adults with hematologic malignancy (HM) are a growing demographic with an increased risk of frailty development [1]. Factors beyond the disease, such as age, comorbidities, and performance status, can impact treatment intensity and tolerability. It is recommended that all adults 65 and older undergo a Geriatric Assessment (GA) to identify occult vulnerabilities that may influence treatment outcomes [2, 3]. A GA more accurately measures health status than clinical judgment alone and can predict mortality and toxicity independent of performance status and age [4–8]. Yet, the adoption and dissemination of routine GAs has proven challenging. As such, hematologists would benefit from rapid and reliable blood-based biomarkers to estimate physiologic age.

Several candidate biomarkers to estimate physiologic age derive from age-related declines in T cell function. These include markers of immunosenescence, exhaustion, and cellular senescence [9–12]. One of the most robust and well-studied markers of cellular senescence, p16^INK4a^ (p16), increases more than 16-fold in peripheral blood T cells over the human lifespan, and higher p16 is associated with biologic aging [13]. Expression of p16 is triggered by cellular stressors such as DNA damage, replication errors, telomere erosion, and reactive oxygen species [14]. p16 is also impacted by lifestyle and environmental factors, such as physical inactivity, chemotherapy, and tobacco exposure [13, 15, 16]. Autologous or allogeneic bone marrow transplant causes dramatic increases in T cell p16 levels and senescence-related gene expression signatures associated with clinical frailty in patients with hematologic malignancies [15, 17]. However, the impact of cancer therapeutics on T cell senescence and physiologic health is unclear. T cell receptor excision circles (TRECs) provide another mechanism to measure age-related changes in T cell production. TRECs are episomal circular DNAs generated during T cell receptor gene rearrangement in the thymus. TRECs are not replicated during proliferation and are therefore diluted among the progeny of naïve T cells [18]. Thus, the ratio of TRECs to T cell genomic DNA is a surrogate for the relative number of circulating naïve T cells [18].

Epigenetic clocks offer a third means of estimating biological age. These algorithms, developed using regression and deep learning methods, define genomic DNA methylation patterns predictive of chronological age and age-related health metrics. Three generations of clocks have been described. First-generation clocks are trained on chronological age and predict mortality better than morbidity [19–21]. Second-generation clocks, like PhenoAge [22] and GrimAge [23] use serum and blood biomarkers to improve morbidity assessment. Distinct from their predecessors, third-generation clocks, such as DunedinPACE [24], capitalize on longitudinal health and DNA methylation data to calculate an instantaneous rate of aging. In patients with HM, the reported effect of hematopoietic stem cell transplant on epigenetic age varies [25–27]. However, where accelerated epigenetic aging is observed, early studies suggest that exercise might partially mitigate these effects [28, 29]. Therefore, epigenetic markers may better identify patient vulnerabilities than chronological age.

In conjunction with clinical frailty assessments, molecular biomarkers of aging may help risk-stratify patients for cancer treatment and identify occult vulnerabilities that could influence clinical outcomes. The primary aim of this pilot study was to benchmark the ability of multiple biomarkers of aging (p16, epigenetic clocks, T cell gene expression profiles, and TRECs) to identify frailty as measured by a clinical impairment index (I^2^) in patients with HM. As a secondary aim, we examined whether these biomarkers were associated with patient outcomes or altered by treatment.

## Methods

### Population and study design

**Fig. 1:**
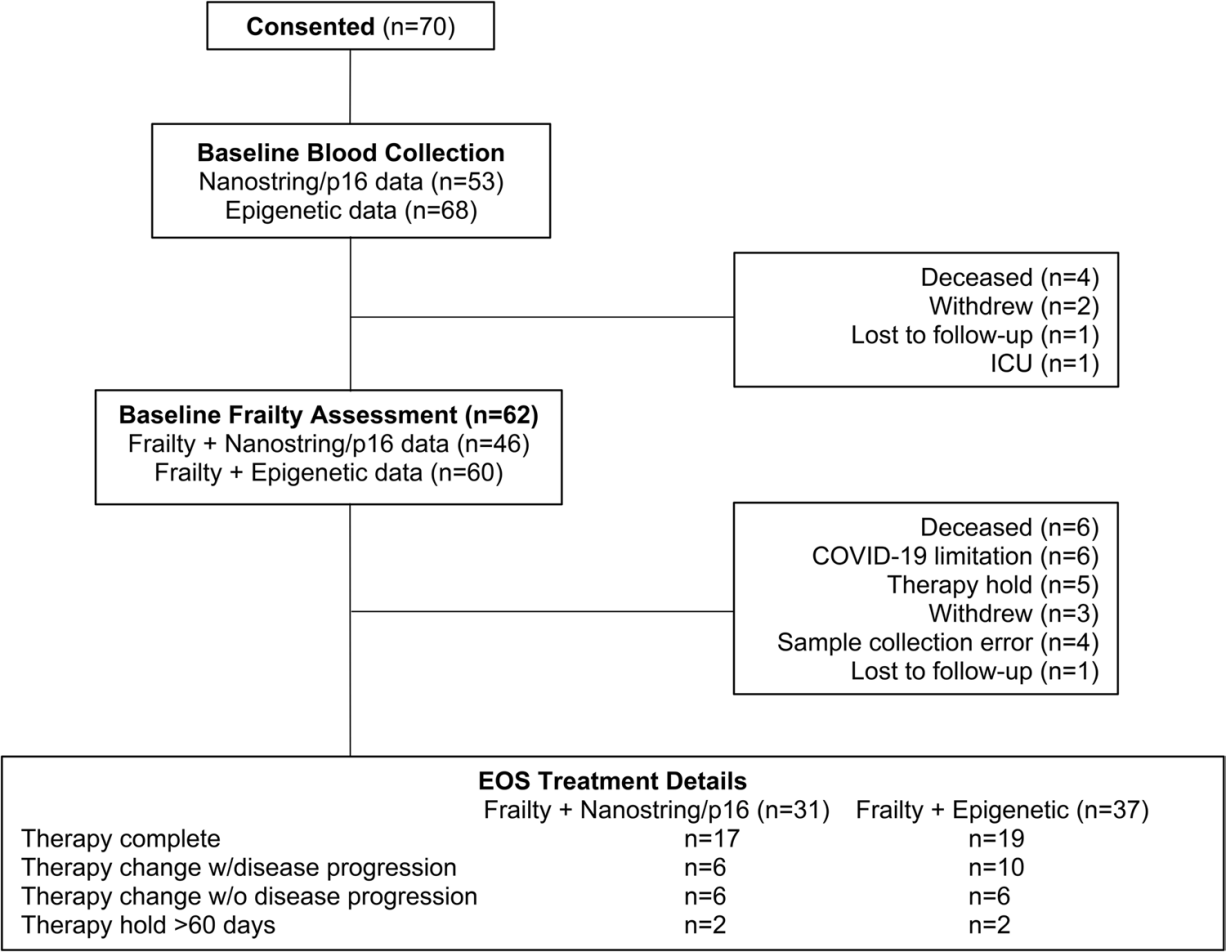
CONSORT Diagram: Flow chart illustrating participant consent, clinical data, and sample procurement

We conducted a single-institution prospective study, approved by The Ohio State University's Institutional Review Board, enrolling 70 patients with HM, and collecting clinical and biomarker data (Fig. 1). Nanostring and epigenetic data were gathered at baseline (pre-treatment for all but 3 samples) and at the End of Study (EOS) from 53 and 33 samples, and 68 and 37 samples, respectively. EOS visits occurred upon chemotherapy completion, disease progression, before stem cell transplant, or after 1 year on study (within 45 days). Additionally, 29 participants without cancer (median age = 47.1; range 22–86 years of age) were recruited from the community as healthy controls, undergoing only initial PBTL p16 and Nanostring profiling.

### Creation of the clinical impairment index (I^2^)

Patients completed a baseline GA as outlined by the Cancer and Aging Research Group (CARG) [8, 30, 31]. GA metrics included Independent Activities of Daily Living (IADLs; [32]), the MOS physical function assessment [33], the MOS social support and activity survey [34], and the Mental Health Inventory (MHI [33];). Performance status was measured using patient-reported Karnofsky Performance Status (KPS) and ECOG performance status scores. A clinical research coordinator administered the Blessed Orientation Memory Concentration (BOMC) cognitive screen [35], and physical function was measured using the Short Physical Performance Battery (SPPB) [36]. Patients received treatment (i.e., chemotherapy, immunotherapy, targeted agents, bone marrow transplant, or other) as ordered by their physician. Medications were enumerated, and information on planned and actual drug dosing collected. Relative Dose Intensity (RDI) was calculated as the ratio of delivered dose intensity to the standard dose intensity [37, 38].

Thresholds for GA impairments were defined and/or adapted from Li et al. [5]. For non-binary variables without established thresholds, we assigned a point for each metric falling within the worst quartile. For example, IADL values ≥ 75th percentile and SPPB values ≤ 25th percentile were each given a point. Patients were assigned one point for each binary metric with a “yes” value. Assigned points from binary and non-binary metrics were combined to generate a single composite impairment index (I^2^) ranging from 0–11. Composite scores were then dichotomized into high and low categories using the cohort’s third quartile value (7.0) as a cutoff.

### p16 and T cell RNA expression profiling

To determine if biologic aging was accelerated at the time of a HM diagnosis, we compared PBTL p16 levels across untreated baseline samples and healthy controls. Peripheral blood (10 ml) was collected in EDTA-coated tubes, and CD3 + T cells isolated via negative selection using RosetteSep reagents. RNA and DNA were extracted from purified PBTLs using the Zymo Research Quick DNA/RNA miniprep kit. T cell RNA quality and quantity were verified on a Bioanalyzer (Agilent) and only samples with an RNA Integrity Number (RIN) of > 7 were used for further analyses. Gene expression was measured using a custom Nanostring CodeSet (OSU_Senescence) comprised of 74 markers of T cell senescence (including p16), function, cytokine production, and differentiation with five housekeeping controls [39]. Nanostring data were normalized to internal controls and across runs using Nanostring nCounter software.

### Epigenetic analysis

DNA from isolated PBTL was sent to TruDiagnostics for epigenetic clock analysis. DNA methylation was measured on Illumina Infinium® MethylationEPIC 850K BeadChips. Raw methylation data was processed using the minfi pipeline and low-quality samples identified using the default parameters of the qcfilter function in the ENmix package. A total of 105 samples passed QA/QC (*p* < 0.05). From these samples, low quality methylation probes (*p* < 0.05 out-of-band) were identified and removed, resulting in 721,802 of 866,239 probes being used for further analysis. The following epigenetic clock algorithms were run using these data: Hannum [40], Horvath 1 (pan-tissue) [20], Horvath 2 (blood and skin) [23], GrimAge [23], PhenoAge [22], AltumAge [41], and DunedinPACE [42]. A combinatorial normalization processing using the Funnorm procedure (minfi package), followed by the RCP method (ENmix package) was performed to minimize sample to sample variation as previously described [42].

### TREC analysis

TREC analysis was completed on the same PBTL DNA used for epigenetic profiling via Taqman-based quantitative real-time PCR with the following primer-probe sets and PerfeCTa FastMic II reagent: hTREC_Forward: 5′-CATCCCTTTCAACCATGCTGACACCTCT-3′; hTREC Reverse: 5′-CGTGAGAACGGTGAATGAAGAGCAGACA-3′; hTREC Probe: 5′-VIC-TTTTTGTAAAGGTGCCCACTCCTGTGCACGGTGA-QSY-3′; hβ-Actin Forward: 5′-TCACCCACACTGTGCCCATCTACGA-3′; hβ-Actin Reverse: 5′-CAGCGGAACCGCTCATTGCCAATGG-3′; hβ-Actin Probe: 5′-FAM-ATGCCCTCCCCCATGCCATCCTGCGT-QSY-3′. Samples were run in technical triplicates on a Bio-Rad CFX Maestro using standard cycling conditions. Relative TREC levels were calculated using the formula: TREC levels = [C_t_ hTREC] – [C_t_ hβ-Actin].

### Statistical analysis

*Primary aim:* The correlations between baseline p16 and each epigenetic clock, TREC, or OSU_Senescence mRNA were examined using Pearson correlation coefficients. Bivariate logistic regression models were used to assess the relationship between baseline I^2^ (outcome) and each biomarker or chronological age (exposures). Youden's J statistics were used to define each biomarker threshold for sensitivity and specificity calculations [43]. The relationship between each aging biomarker and baseline I^2^ was calculated with and without adjustments for chronological age and disease groups. To determine how each aging biomarker improved frailty discrimination, we used DeLong tests to compare the AUCs of each biomarker adjusted for chronological age and disease group to the AUCs of a model that included only chronological age and disease group.

*Secondary aims:* Descriptive statistics were used to summarize baseline characteristics for all patients and for those with complete p16 data. Median and interquartile ranges were provided for continuous variables, and frequency and percentage calculated for categorical variables. Age-adjusted p16 levels were estimated using linear regression models. Specifically, we fit linear regression models using p16 as the outcome with 1) chronological age and disease groups or 2) chronological age and chemotherapy intensity groups as independent variables. In addition, we examined the age-adjusted treatment-related changes in p16 for patients whose cancer was controlled by the of end of the study. In a posthoc analysis, we further adjusted our linear regression models testing treatment-related changes in p16 to account for a potential effect of length of follow-up. We also used linear regression models to examine the association between p16 and RDI, adjusting for chronological age, disease, and treatment groups. In this analysis, a significance level of 0.05 for two-sided tests was considered statistically significant. All analyses were performed using SAS version 9.4 and R version 4.2.0.

## Results

### Sample cohort

The mean age of the study population was 71.1 [standard deviation (SD) = 7.0] and the mean baseline p16 level was 50.7 (SD = 65.6) (Table 1). Treatment was variable and included targeted (44.3%), high-dose multi-drug (29.5%), hypomethylating and targeted (13.1%), low-dose multi-drug (9.8%), and hypomethylating only regimens (3.3%) (Supplemental Table 1).

**Table 1:** Study sample characteristics

Variable	All (*n* = 70)	With P16^INK4a^ data (*n* = 53)
Baseline	70	53
End of study	37	31
Baseline chronologic age		
Mean (SD)	71.1 (7.0)	70.9 (6.8)
Median (25th, 75th percentile)	70.2 (65.2, 76.4)	70.2 (65.3, 75.4)
Sex, n (%)		
Male	43 (61.4)	30 (56.6)
Female	27 (38.6)	23 (43.4)
Race, n (%)		
White	68 (97.1)	51 (96.2)
Other	2 (2.9)^a^	2 (3.8)^a^
Disease group, n (%)		
Acute leukemia or Myelodysplastic syndrome	30 (42.9)	20 (37.7)
Chronic lymphocytic leukemia	5 (7.1)	3 (5.7)
Lymphoma	15 (21.4)	13 (24.5)
Plasma cell disorder (myeloma, amyloid)	20 (28.6)	17 (32.1)
Chemotherapy intensity, n (%)		
High-dose multi-drug	18 (29.5)	16 (34.8)
Hypomethylating	2 (3.3)	2 (4.4)
Hypomethylating, targeted	8 (13.1)	4 (8.7)
Low-dose multi-drug	6 (9.8)	4 (8.7)
Targeted	27 (44.3)	20 (44.5)
Relative Dose Intensity (RDI)		
Mean (SD)	75.7 (34.6)	78.3 (31.8)
Median (25th, 75th percentile)	90.7 (70.1, 100.0)	92.2 (75.0, 100.0)
P16^INK4a^	Baseline (*n* = 53)	End of Study (*n* = 31)
Mean (SD)	50.7 (65.6)	67.5 (104.9)
Median (25th, 75th percentile)	67.5 (104.9)	33.8 (14.9, 98.1)

*SD* standard deviation

^a^One patient identified as African American, and one patient as American Indian/Alaskan Native

### Relationship of PBTL p16 levels with diagnosis and clinical outcomes

**Fig. 2:**
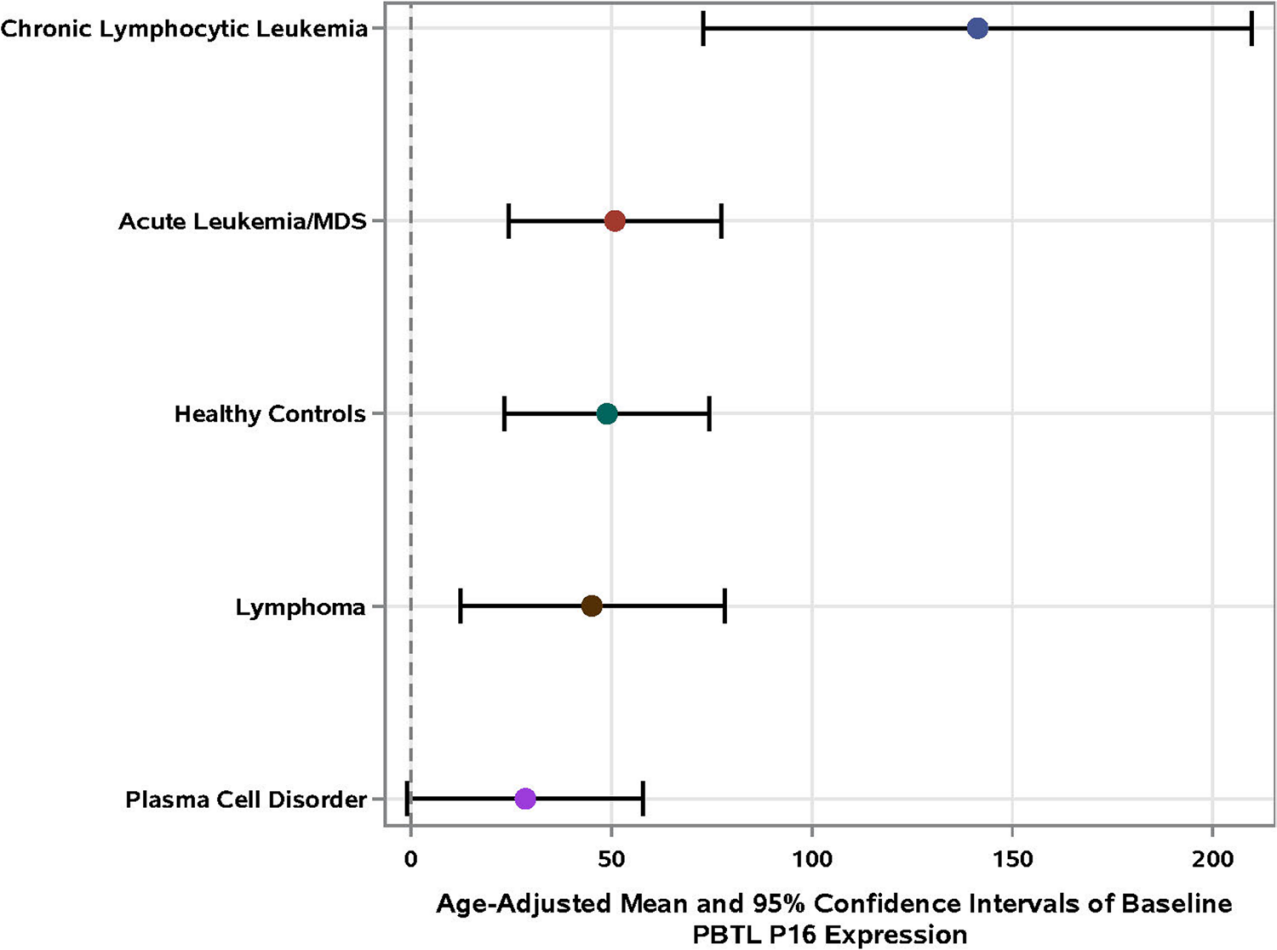
Comparison of mean PBTL p16 levels [and 95% confidence intervals (CI)] by cancer type

The mean baseline age-adjusted p16 was similar among patients and healthy controls (*p* > 0.11). However, the three patients with chronic lymphocytic leukemia [mean = 141.3, 95% CI (107.0–175.7)] had significantly higher baseline age-adjusted p16 levels than those with plasma cell disorders [mean = 28.4, 95% CI (13.7–43.2) *p* = 0.02]. A comparison of mean PBTL p16 levels [and 95% confidence intervals (CI)] by cancer type and of healthy controls are shown in Fig. 2. We also examined whether baseline p16 differed among patients who died during the study period (1 year; *n* = 10) versus those who did not (*n* = 46) and found no statistically significant difference (Supplemental Fig. 1).

### Differences in PBTL p16 levels at baseline, with treatment, and RDI

The average p16 level was 50.7 (SD = 65.6) at baseline and increased to 67.5 (SD = 104.9) at EOS (*p* = 0.43). Treatment-related changes in p16 were evaluated in 19 patients with disease control (therapy complete or therapy changed without disease progression) at EOS (N = 19). Therapy duration averaged 135.2 (median = 72.3) days with minimal difference between treatment groups [high-dose multi-drug median = 140.0 (interquartile range (IQR) 92.0–158.0), low-dose multi-drug median = 288.5 (IQR 273.0–304.0), hypomethylating with targeted therapy median = 129.0 (IQR 123.0–135.0), targeted therapy only median = 133.5 (IQR 91.0- 366.0)]. None of the therapies induced statistically significant changes in age-adjusted p16 among this small subset of patients (Supplemental Fig. 2). However, patients who received targeted therapy showed the greatest increase in p16 relative to baseline [mean = 0.40, 95% CI (-0.97–1.78)] whereas patients receiving a combination of hypomethylating and targeted agents had the largest decrease [mean = -1.09, 95% CI (-3.47–1.29)]. These results remained largely unchanged when adjusted for the length of follow-up. Among the entire cohort, baseline p16 levels did not significantly correlate with RDI [r = 0.15, 95% CI (-0.15–0.42)]. However, a unit increase in baseline p16 was significantly associated with an increase in mean RDI of 0.17 (95% CI 0.05 – 0.29) when adjusting for chronological age, disease, and treatment groups.

### Relationship between PBTL p16 and other aging biomarkers

**Fig. 3:**
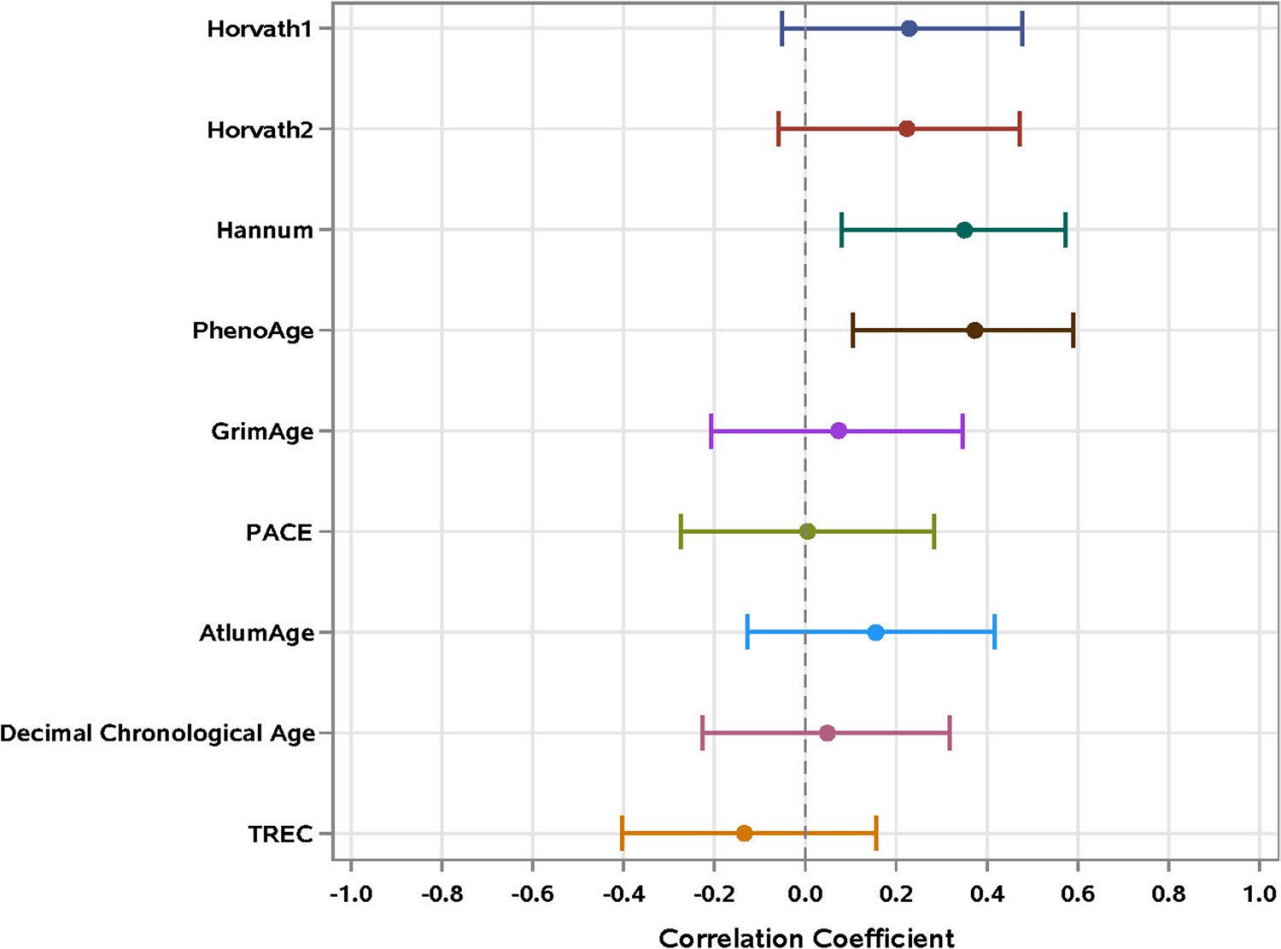
Correlations [95% confidence intervals (CI)] of baseline PBTL p16 with chronological age and other aging biomarkers. PACE=Dunedin PACE, TREC= T cell receptor excision circles

We next examined the relationship between baseline p16 and multiple aging indicators. p16 levels did not correlate with chronologic age in our HM cohort [r = 0.04, 95% CI (-0.24–0.30); p adj. = 0.90). However, p16 correlated positively with the Hannum [r = 0.35, 95% CI (0.09–0.57); p adj. = 0.04] and PhenoAge [r = 0.37, 95% CI (0.11–0.59); p adj. = 0.04] clocks (Fig. 3). TRECs did not correlate with p16, suggesting that the relative frequency of circulating naive T cells was not related to PBTL cellular senescence in patients with HM [r = -0.13, 95% CI (-0.40–0.16); *p* = 0.54]. In comparisons between baseline p16 and OSU_Senescence Nanostring values, the most significantly correlated markers (*p* ≤ 0.005) included mRNAs indicative of cellular senescence (*Cdkn2A ARF*, *B3gat1* (CD57), *Il-6*), potential T cell exhaustion (*Cd244*, *Cd276*, *Btla*, *Pdcd1*, *Pdcd1lg2*, *Pvr*), T follicular helper cells (*Bcl-6*, *Il-21*), and terminally differentiated effector memory populations (*Eomes*) (Supplemental Fig. 3; Complete statistics provided in Supplemental Table 2).

### P16 as an indicator of clinical frailty in hematological malignancies

Nearly three quarters (73.3%) of study participants exhibited functional impairment as measured by the SPPB, and over half of patients (58.1%) had iADLS deficits at baseline. Self-reported performance status was worse than physician-reported performance measures [self-reported KPS impairment (53.2%), ECOG impairment (26.7%)]. Polypharmacy was present in over half of patients (62.9%), and patients were well supported but not socially active as measured by the MOS social support scales (Table 2). Individual geriatric metrics were not significantly associated with p16 levels at baseline or when adjusted by age and disease group (Supplemental Table 3).

**Table 2:** Composite Impairment Index (I^2^) with included individual metric thresholds*

Metric	Score	Definition of Impairment	Number of Patients with Impairment (%)	Mean (SD)	Median (Q1, Q3)
Impairment Index (I^2^)	Range 0–11	≥ 7	13/50 (26.0%)	5.2 (2.3)	6.0 (4.0 to 7.0)
Instrumental activities of daily living (IADLs)	Range 0–14	< 14	36/62 (58.1%)	11.9 (2.5)	13.0 (10.0 to 14.0)
SPPB	Range 0–12	< 9	44/60 (73.3%)	5.1 (5.1)	5.0 (0.0 to 11.0)
Physical Health Scale – OARS subscale	Range 0–22	≥ 3	59/62 (95.2%)	5.7 (1.9)	6.0 (4.0 to 7.0)
Body Mass Index (BMI)	Kg/m^2^	Impaired < 18.5 or ≥ 30 kg/m^2^	31/69 (44.9%)	31.1 (6.8)	29.1 (26.1 to 35.0)
ECOG PS	Range 0–4	≥ 2	16/60 (26.7%)	1.2 (0.7)	1.0 (1.0 to 2.0)
Self reported-KPS	Range 30–100	< 80	33/62 (53.2%)	72.4 (18.2)	70.0 (60.0 to 90.0)
Blessed Orientation Memory Concentration	Range 0–28	≥ 11	2/62 (3.2%)	3.7 (3.4)	3.0 (0.0 to 6.0)
Polypharmacy	Range 0–14	≥ 5 Medications	39/62 (62.9%)	5.9 (3.6)	5.0 (3.0 to 8.0)
Mental Health Inventory	Yes/No	Response of “all of the time” or “a good bit of the time” to questions affirming anxiety or depression	24/62 (38.7%)	N/A	N/A
Social Activity Score	Yes/No	Response to queries*	44/62 (71.0%)	N/A	N/A
Social Support Score	Yes/No	Response of “none of the time” or “a little of the time”	9/62 (14.5%)	N/A	N/A

*SPPB* Short Physical Performance Battery, *OARS* Older Americans Resources and Services, *ECOG* Eastern Cooperative Oncology Group performance status, *KPS* Karnofsky Performance Status, *SD* Standard Deviation

^*^Response to questions indicate at least one of the following: “all of the time” to “some of the time” on interference of physical health or emotional problems on social activities—“much less socially active than before” in the past 6 months—“much more limited than others” compared to individuals who are the same age. *I^2^ scoring criteria and model described in Methods, impairment thresholds adopted and modified from Li E et al. [4]

When adjusting for chronologic age and disease group, four aging biomarkers showed significant ability to identify impairment, as defined by an Impairment Index (I^2^) score of 7 or more: p16^INK4a^ [AUC = 0.76, 95% CI (0.56–0.98); *p* = 0.01], Hannum [AUC = 0.70, 95% CI (0.54–0.85); *p* = 0.01], PhenoAge [AUC = 0.71, 95% CI (0.55–0.86); *p* = 0.01], and DunedinPACE [AUC = 0.73, 95% CI (0.57–0.88); *p* =  < 0.01] (Table 3). Neither chronologic age (AUC = 0.58), TREC values, nor the Horvath, GrimAge, and AtlumAge clocks, showed significant potential to identify impairment (Table 3). To test whether each aging biomarker improved frailty discrimination by chronological age and disease group, we performed DeLong tests comparing the AUCs of each biomarker adjusted for chronological age and disease group to an AUC model that included only chronologic age and disease group. Though not statistically significant, p16 (*p* = 0.16) and PhenoAge (*p* = 0.15) led to the most dramatic improvements in I^2^ discrimination among aging biomarkers measured in this limited pilot cohort.

**Table 3:** Area Under the Curve (AUC) for the crude and adjusted associations between selected aging biomarkers and the composite Impairment Index (I^2^)

Aging biomarkers	Crude^a^		Adjusted^b^	
	AUC (95% CI)	*P* Value	AUC (95% CI)	*P* Value
p16	0.63 (0.39 to 0.87)	0.28	0.76 (0.56 to 0.98)	0.01
Horvath1	0.46 (0.27 to 0.64)	0.64	0.66 (0.49 to 0.83)	0.06
Horvath2	0.48 (0.28 to 0.68)	0.83	0.67 (0.50 to 0.83)	0.05
Hannum	0.52 (0.32 to 0.72)	0.86	0.70 (0.54 to 0.85)	0.01
PhenoAge	0.54 (0.33 to 0.75)	0.68	0.71 (0.55 to 0.86)	0.01
GrimAge	0.49 (0.28 to 0.70)	0.90	0.64 (0.47 to 0.82)	0.11
PACE	0.49 (0.30 to 0.70)	0.98	0.73 (0.57 to 0.88)	< 0.01
AtlumAge	0.51 (0.30 to 0.71)	0.96	0.66 (0.49 to 0.82)	0.06
TREC	0.52 (0.30 to 0.74)	0.88	0.65 (0.47 to 0.84)	0.11

TREC = T cell receptor excision circles

^a^ For the bivariate association between each aging biomarker and the composite Impairment Index

^b^ For the association between each aging biomarker and the Composite Impairment Index adjusted for chronologic age and disease group

## Discussion

Several factors, including comorbidities, reduced functional reserves, and increased susceptibility to treatment-related toxicities, complicate the treatment of older adults with HM. While the evidence is clear that a complete GA can identify occult vulnerabilities and improve clinical outcomes [3–5], such measures are not always clinically feasible nor routinely implemented [44]. Blood-based aging biomarkers could facilitate the identification of at-risk individuals and aid in therapeutic decision-making. Multiple aging biomarkers have emerged in past decades. However, each marker has limitations in sensitivity and may be impacted by underlying diseases like cancer. Here, we measured p16, T cell mRNAs, TRECs, and seven different epigenetic clocks in patients with comprehensive geriatric profiles using validated tools. Our goal was to assess the ability of PBTL p16 to identify clinical impairment and to benchmark this key aging biomarker against other biomarkers in the field. We discovered potential relationships between PBTL p16 levels and two epigenetic clocks (i.e., Hannum and PhenoAge), as well as multiple mRNA markers of T cell senescence. We created a new tool, the I^2^ index, to quantify and set thresholds for impairment across geriatric domains built from an evidence-based approach. Using this tool, we determined that among the aging biomarkers measured, p16 and PhenoAge had the greatest potential to improve frailty detection beyond chronologic age and disease type in patients with HM.

PBTL p16 levels did not differ between patients with HM and healthy controls at diagnosis but were correlated with other markers of T cell senescence and aging. These data suggest that PBTL senescence is generally not accelerated among untreated patients with HM. This finding is consistent with that of Wood et al., who saw no difference in PBTL p16 among newly diagnosed and pre-treated patients with distinct HM [17]. Prior studies reported that PBTL p16 levels increased among patients receiving high-dose chemotherapy whereas therapy had no significant effect on PBTL p16 in our dataset [16, 45]. Several factors likely hindered our ability to detect treatment-related PBTL p16 increases, including the limited number of paired samples acquired due to COVID-19 restrictions and other events, therapeutic diversity even within the same treatment group, and changes in disease burden since all the patients analyzed were responders. This diversity in the patient population and therapeutic regimens emphasizes the need for consistent approaches for estimating vulnerability and standardizing treatment in older adults with HM.

In our cohort, PBTL p16 levels correlated most closely with the Hannum and PhenoAge clocks. Interestingly, PhenoAge was one of the first clocks trained to predict mortality based on a combination of clinical lab metrics (albumin, creatinine, C-reactive protein, etc.) and chronologic age [22]. PhenoAge also correlates with the ratio of naïve, CD8 to CD4 T cells, suggesting a relationship between this clock and immunosenescence [46]. Notably, the relationship between PBTL p16 and the Horvath 1 clock (r = 0.23) was less robust than in our prior analysis of healthy individuals over 40 years of age (r = 0.82 [39],), suggesting that a HM diagnosis may decrease the contribution of chronologic age to PBTL p16 levels. Whether a novel epigenetic clock could better estimate the relative contributions of chronologic and physiologic aging to PBTL p16 levels is unclear. However, as discussed below, such a metric could overcome some technical challenges associated with measuring p16 in broad clinical settings.

We focused our analysis on CD3 + PBTLs as p16 increases most dramatically in this subset of peripheral blood cells [13]. However, CD3 + PBTL are a mixture of functionally diverse subsets that change with age. Leveraging the OSU_Senescence Nanostring platform, we gained a deeper understanding of the relationship between p16 and CD3 + T cell subsets. Our analyses revealed robust correlations between p16 and PBTL mRNAs associated with cellular senescence (*Cdkn2a_ARF*, *B3gat1* (CD57), *Il-6*), exhaustion (*Cd244*, *Cd276*, *Btla*, *Pdcd1* (PD-1), *Pdcd1lg2* (PDL-2), *Pvr*), T follicular helper cells (*Bcl-6*, *Il-21*), and terminally differentiated effector memory populations (*Eomes*). Correlations with markers of the senescence-associated secretory phenotype (i.e., *Il-6*), reduced proliferative potential (*B3gat1*), and terminally differentiated effector T cells are consistent with the idea that PBTL p16 measures age-related T cell phenotypes, including cellular and immunosenescence. In other studies of HM, a positive correlation between PBTL *p16* and *Cd244* was observed (r = 0.284, *p* = 0.008 [39]. Whether CD244 could serve as a surrogate for PBTL p16 expression is unknown. However, its expression alongside other markers of exhaustion in patients with cancer [47] suggests an association with age-related T cell dysfunction that should be explored in the future.

Several clinical tools are used to characterize frailty in cancer, including a number that are specific to HM. The clinical impairment index (I^2^) we describe is a comprehensive tool with defined thresholds of impairment for each domain. This equips clinicians with a practical means to identify vulnerabilities across geriatric domains. Our work builds upon the Practical Geriatric Assessment (PGA) [2], recommended for all older adults with cancer, by summarizing deficits into a single score. Importantly, the I^2^ and defined thresholds for geriatric metrics, will need to be validated in future studies. In this cohort of older adults with HM, patients exhibited significant clinical impairment at the time of diagnosis, emphasizing the importance of identifying and intervening on age-related deficits, particularly in high acuity illnesses like HM. When adjusting for chronologic age and disease group, we defined thresholds of p16 that identifies frailty as measured by the clinical impairment index (I^2^). In addition, baseline p16, when adjusted by age and disease, was predictive of chemotherapy tolerance, as measured by RDI. Upfront treatment dose attenuations are often based on organ impairment (i.e. renal or liver function abnormalities) or perception of poor treatment tolerance. Biomarkers of aging, like p16, may aid in identifying physiologic health and could serve as a more reliable indicator of treatment tolerance. Although p16 analysis has limitations, future studies integrating blood-based biomarkers to augment frailty assessments, may provide valuable insight on patient trajectories.

This report is a pilot study, which requires validation in a larger cohort where changes in p16 expression with treatment and disease control can be better evaluated. The durability of increased PBTL p16 expression also requires further examination, although our prior data show that increases in p16 are sustained long term [15]. Most epigenetic clocks were developed using whole blood. However, our study used purified PBTL so that p16 and epigenetic age could be assessed in the same sample. We have seen a direct correlation between epigenetic clocks measured in blood or PBTLs from the same healthy donor [48], but recognize that PBTL-specific features and the lack of age-correction are limitations to the interpretation of these data. Despite these limitations, we successfully implemented a panel of aging biomarkers in a high-acuity cancer population, addressing technical challenges. For example, p16 levels differ based on cell type [13], making it necessary to isolate specific peripheral blood cell subsets on site. Equipment and trained staff for isolation are often lacking, and shipping samples offsite can lead to RNA degradation. Epigenetic clocks and TRECs are more stable but face barriers associated with cost and availability. One solution would be to create algorithms to estimate one measurement from another, but this will likely require further biotech investments to reduce cost, standardize assessments, and improve availability.

In summary, our pilot data suggest that molecular markers of aging, particularly PBTL p16 and PhenoAge, have the potential to characterize frailty in older adults with HM. Further research is needed to validate the utility of these and other molecular markers in larger cohorts and different cancer populations. Integrating molecular markers of aging into clinical practice could lead to more personalized and effective treatment approaches in this vulnerable patient population. We are actively refining our predictive model by combining multiple aging biomarkers, aiming to capture aging more comprehensively and optimize the care of vulnerable cancer populations.

### Supplementary Information

Below is the link to the electronic supplementary material.

Supplementary file1 (DOCX 40 KB)

Supplementary file2 (JPG 106 KB)

Supplementary file3 (JPG 113 KB)

Supplementary file4 (JPG 549 KB)
