# Supplementary material for: Determining the relationship of p16INK4a and additional molecular markers of aging with clinical frailty in hematologic malignancy
Source: J Cancer Surviv. 2024 Apr 28;18(4):1168–78. doi: 10.1007/s11764-024-01591-6 (PMC11324703; doi:10.1007/s11764-024-01591-6)
Supplement: Supplementary file 1 — Supplementary file1 (DOCX 40 KB) [file 11764_2024_1591_MOESM1_ESM.docx]

| **Supplemental Table 1. Chemotherapy Classification** | | |
| --- | --- | --- |
| **Disease Group** | **Chemotherapy Category** | **Chemotherapy Regimen** |
| Acute Leukemia | High-dose/multi-drug | cyclophosphamide, daunorubicin, vincristine, pegaspargase, cytarabine, methotrexate (STOCK) (n=1) |
|  |  | daunorubicin-cytarabine liposome (n=1) |
|  |  | E-selection antagonist, daunorubicin, cytarabine (n=1) |
|  |  | rituximab, dexamethasone, cytarabine, daunorubicin, vincristine, methotrexate (n=1) |
|  |  | rituximab, cyclophosphamide, vincristine sulfate, prednisone (RCVP) (n=1) |
|  | Hypomethylating | azacitidine (n=2) |
|  | Hypomethylating/Targeted | CD33 monoclonal antibody, azacitidine (n=2) |
|  |  | entospletinib, decitabine (n=1) |
|  |  | ivosidenib, azacitidine (n=2) |
|  |  | sorafenib, azacitidine (n=1) |
|  |  | venetoclax, azacitidine (n=2) |
|  | Targeted | enasidenib (n=5) |
|  |  | entospletinib (n=1) |
|  |  | gilteritinib (n=1) |
| CLL | Targeted | ibrutinib , obinutuzumab (n=2) |
|  |  | rituximab (n=2) |
| Lymphoma | High-dose/multi-drug | adriamycin, bleomycin, vinblastine, dacarbazine (ABVD) (n=1) |
|  |  | brentuximab vedotin, doxorubicin, vinblastine, dacarbazine (AVD+BV) (n=1) |
|  |  | DA-REPOCH, nivolumab (n=1) |
|  |  | obinutuzumab, methotrexate (n=1) |
|  |  | R-CHOP (n=2) |
|  |  | REPOCH (n=4) |
|  |  | RM-CHOP (n=2) |
|  |  | R-miniCHOP (n=1) |
|  | Low-dose/multi-drug | rituximab, bendamustine (n=1) |
| Plasma Cell Disease | Low-dose/multi-drug | cyclophosphamide, bortezomib, dexamethasone, rituximab (CyBorD + rituximab) (n=1) |
|  |  | cyclophosphamide, bortezomib, dexamethasone (CyBorD) (n=4) |
|  | Targeted | bortezomib, dexamethasone (n=6) |
|  |  | bortezomib, lenalidomide, dexamethasone (VRd) (n=) |
|  |  | pevonedistat, azacitidine (n=1) |
|  |  | daratumamab, lenalinomide (n=1) |

| **Supplemental Table 2. OSU_Senescence Nanostring** | | | | | | | | | |
| --- | --- | --- | --- | --- | --- | --- | --- | --- | --- |
| Var | NObs | Corr | ZVal | BiasAdj | CorrEst | Lcl | Ucl | Raw P Values | **False**  **Discovery**  **Rate Corrected P values** |
| ADORA2A | 53 | 0.45317 | 0.48868 | 0.00436 | 0.4497 | 0.204227 | 0.641961 | 0.000549322 | 0.0009 |
| B3GAT1 | 53 | 0.48014 | 0.52316 | 0.00462 | 0.47658 | 0.236785 | 0.661641 | 0.000216179 | 0.0004 |
| BCL6 | 53 | 0.50818 | 0.56028 | 0.00489 | 0.50455 | 0.271246 | 0.681854 | 7.44031E-05 | 0.0003 |
| BTLA | 53 | 0.62942 | 0.74045 | 0.00605 | 0.62575 | 0.427814 | 0.766415 | 1.64291E-07 | <.0001 |
| C10orf54 | 53 | 0.35032 | 0.36581 | 0.00337 | 0.34736 | 0.085052 | 0.56464 | 0.00969152 | 0.0119 |
| CCR4 | 53 | 0.30628 | 0.31644 | 0.00295 | 0.30361 | 0.036296 | 0.53038 | 0.025250325 | 0.0279 |
| CCR6 | 53 | 0.50029 | 0.54969 | 0.00481 | 0.49667 | 0.261483 | 0.67619 | 0.000101531 | 0.0003 |
| CD127 | 53 | -0.27538 | -0.28267 | -0.00265 | -0.27293 | -0.5059 | -0.00284 | 0.045630803 | 0.0469 |
| CD160 | 53 | 0.4444 | 0.4777 | 0.00427 | 0.44096 | 0.193765 | 0.635511 | 0.000730548 | 0.0012 |
| CD200R1 | 53 | 0.39333 | 0.41573 | 0.00378 | 0.39012 | 0.133956 | 0.597421 | 0.003285906 | 0.0043 |
| CD244 | 53 | 0.51189 | 0.56529 | 0.00492 | 0.50825 | 0.275853 | 0.684509 | 6.40931E-05 | 0.0003 |
| CD276 | 53 | 0.55646 | 0.62769 | 0.00535 | 0.55276 | 0.332079 | 0.716066 | 9.06013E-06 | <.0001 |
| CD80 | 53 | 0.53708 | 0.60004 | 0.00516 | 0.53339 | 0.307421 | 0.702417 | 2.20631E-05 | 0.0001 |
| CD86 | 53 | 0.48365 | 0.52773 | 0.00465 | 0.48008 | 0.241063 | 0.664184 | 0.000190237 | 0.0004 |
| CD96 | 53 | -0.27312 | -0.28023 | -0.00263 | -0.27069 | -0.5041 | -0.00042 | 0.047531607 | 0.0475 |
| CDKN2A_ARF | 53 | 0.77868 | 1.04201 | 0.00749 | 0.77571 | 0.639506 | 0.864705 | 1.73195E-13 | <.0001 |
| CEACAM1 | 53 | 0.55621 | 0.62733 | 0.00535 | 0.5525 | 0.331754 | 0.715888 | 9.17001E-06 | <.0001 |
| CXCR3 | 53 | 0.30533 | 0.31538 | 0.00294 | 0.30266 | 0.035251 | 0.529627 | 0.025741662 | 0.0279 |
| EGR1 | 53 | 0.44322 | 0.47624 | 0.00426 | 0.43979 | 0.192366 | 0.634644 | 0.000758541 | 0.0012 |
| EOMES | 53 | 0.4834 | 0.52741 | 0.00465 | 0.47983 | 0.240762 | 0.664006 | 0.000191964 | 0.0004 |
| GRAIL | 53 | 0.45982 | 0.49708 | 0.00442 | 0.45633 | 0.212206 | 0.646837 | 0.000439914 | 0.0008 |
| HAVCR2 | 53 | 0.43358 | 0.46429 | 0.00417 | 0.43018 | 0.180928 | 0.627513 | 0.001026955 | 0.0015 |
| IFNG | 53 | 0.32434 | 0.33649 | 0.00312 | 0.32155 | 0.056135 | 0.544518 | 0.017342302 | 0.0200 |
| IL_10 | 53 | 0.45604 | 0.4923 | 0.00438 | 0.45256 | 0.207669 | 0.644069 | 0.000499385 | 0.0009 |
| IL_2 | 53 | 0.41287 | 0.43906 | 0.00397 | 0.40957 | 0.156613 | 0.612101 | 0.001905071 | 0.0026 |
| IL_21 | 53 | 0.49034 | 0.5365 | 0.00471 | 0.48675 | 0.249244 | 0.669021 | 0.000148452 | 0.0004 |
| IL_4 | 53 | 0.33183 | 0.34488 | 0.00319 | 0.32899 | 0.06442 | 0.550342 | 0.014741139 | 0.0175 |
| IL6 | 53 | 0.59034 | 0.67819 | 0.00568 | 0.58663 | 0.37595 | 0.739645 | 1.62238E-06 | <.0001 |
| KIR_Activating_Subgroup_1 | 53 | 0.46434 | 0.50283 | 0.00446 | 0.46083 | 0.217643 | 0.650141 | 0.000377233 | 0.0007 |
| KIR_Activating_Subgroup_2 | 53 | 0.38868 | 0.41025 | 0.00374 | 0.3855 | 0.128612 | 0.593913 | 0.003721201 | 0.0047 |
| KIR_Inhibiting_Subgroup_1 | 53 | 0.49481 | 0.54241 | 0.00476 | 0.49121 | 0.254739 | 0.672249 | 0.000125341 | 0.0004 |
| KIR_Inhibiting_Subgroup_2 | 53 | 0.48151 | 0.52495 | 0.00463 | 0.47795 | 0.238458 | 0.662637 | 0.000205669 | 0.0004 |
| PDCD1 | 53 | 0.58638 | 0.67213 | 0.00564 | 0.58266 | 0.370762 | 0.736903 | 2.00774E-06 | <.0001 |
| PDCD1LG2 | 53 | 0.50268 | 0.55288 | 0.00483 | 0.49905 | 0.264431 | 0.677905 | 9.25049E-05 | 0.0003 |
| PVR | 53 | 0.4994 | 0.54851 | 0.0048 | 0.49579 | 0.260391 | 0.675553 | 0.00010508 | 0.0003 |
| RORC | 53 | 0.48228 | 0.52595 | 0.00464 | 0.47871 | 0.239396 | 0.663194 | 0.000199986 | 0.0004 |
| TNFRSF18 | 53 | 0.27818 | 0.28571 | 0.00267 | 0.27571 | 0.005851 | 0.508135 | 0.043357628 | 0.0458 |
| VTCN1 | 53 | 0.41396 | 0.44038 | 0.00398 | 0.41066 | 0.15789 | 0.612919 | 0.001845807 | 0.0026 |

| **Supplemental Table 3. Relationship of individual geriatric metrics with p16** | | | | | |  |  |  |  |  |
| --- | --- | --- | --- | --- | --- | --- | --- | --- | --- | --- |
| **Metric** | **Score** | **Definition of Impairment** | **Number of Patients (%)** | **Crude** | **Adjusted*** |  |  |  |  |  |
|  |  |  |  | **OR (95% CI)** | **OR (95% CI)** |  |  |  |  |  |
| **Instrumental activities of daily living (IADLs)** | Range 0-14 | < 14 | 36/62 (58.1%) | 1.00 (0.99 to 1.01) | 1.00 (0.99 to 1.01) |  |  |  |  |  |
| **SPPB** | Range 0-12 | < 9 | 44/60 (73.3%) | 1.00 (0.99 to 1.01) | 1.00 (0.99 to 1.01) |  |  |  |  |  |
| **Physical Health Scale – OARS subscale** | Range 0-22 | ≥ 3 | 59/62 (95.2%) | 0.99 (0.98 to 1.01) | 1.00 (0.98 to 1.01) |  |  |  |  |  |
| **Body Mass Index (BMI)** | Kg/m^2^ | Impaired <18.5 or ≥ 30 Kg/m^2^ | 31/69 (44.9%) | 1.00 (0.99 to 1.01) | 1.00 (0.99 to 1.01) |  |  |  |  |  |
| **ECOG** | Range 0-4 | ≥ 2 | 16/60 (26.7%) | 1.00 (0.99 to 1.01) | 1.00 (0.98 to 1.01) |  |  |  |  |  |
| **Self Reported-KPS** | Range 30-100 | < 80 | 33/62 (53.2%) | 1.00 (0.99 to 1.01) | 1.00 (0.99 to 1.01) |  |  |  |  |  |
| **Polypharmacy** | Range 0-14 | ≥ 5 Medications | 39/62 (62.9%) | 1.00 (0.99 to 1.01) | 1.00 (0.99 to 1.01) |  |  |  |  |  |
| **Mental Health Inventory** | Yes/No | Response of “all of the time” or “a good bit of the time” to questions affirming anxiety or depression | 24/62 (38.7%) | 1.00 (0.99 to 1.01) | 1.00 (0.98 to 1.01) |  |  |  |  |  |
| **Social Activity Score** | Yes/No | Response to queries* | 44/62 (71.0%) | 1.00 (0.99 to 1.01) | 1.00 (0.99 to 1.01) |  |  |  |  |  |
| **Social Support Score** | Yes/No | Response of “none of the time” or “a little of the time” | 9/62 (14.5%) | 1.00 (0.98 to 1.01) | 1.00 (0.98 to 1.01) |  |  |  |  |  |
| * Adjusted for chronologic age and disease group  OR = Oddis ratio, CI = Confidence Interval | | | | | |  |  |  |  |  |
|  | | | | | |  |  |  |  |  |
|  | | | | | |  |  |  |  |  |
|  |  |  |  |  |  |  |  |  |  |  |
