# Supplementary figures and images for: Determining the relationship of p16INK4a and additional molecular markers of aging with clinical frailty in hematologic malignancy
Source: J Cancer Surviv. 2024 Apr 28;18(4):1168–78. doi: 10.1007/s11764-024-01591-6 (PMC11324703; doi:10.1007/s11764-024-01591-6)

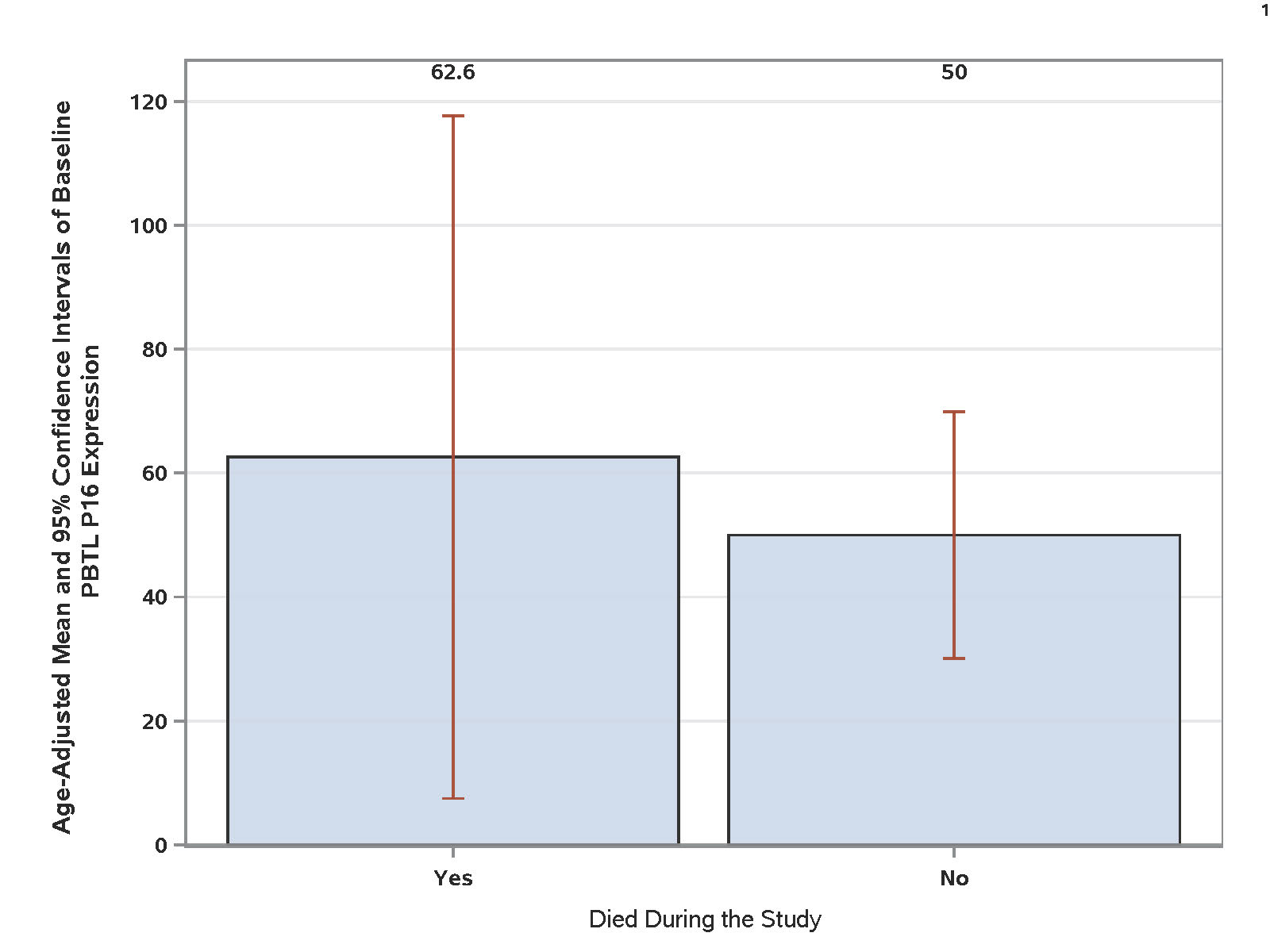

Supplement: Supplementary file 2 — Supplementary file2 (JPG 106 KB) [file 11764_2024_1591_MOESM2_ESM.jpg]

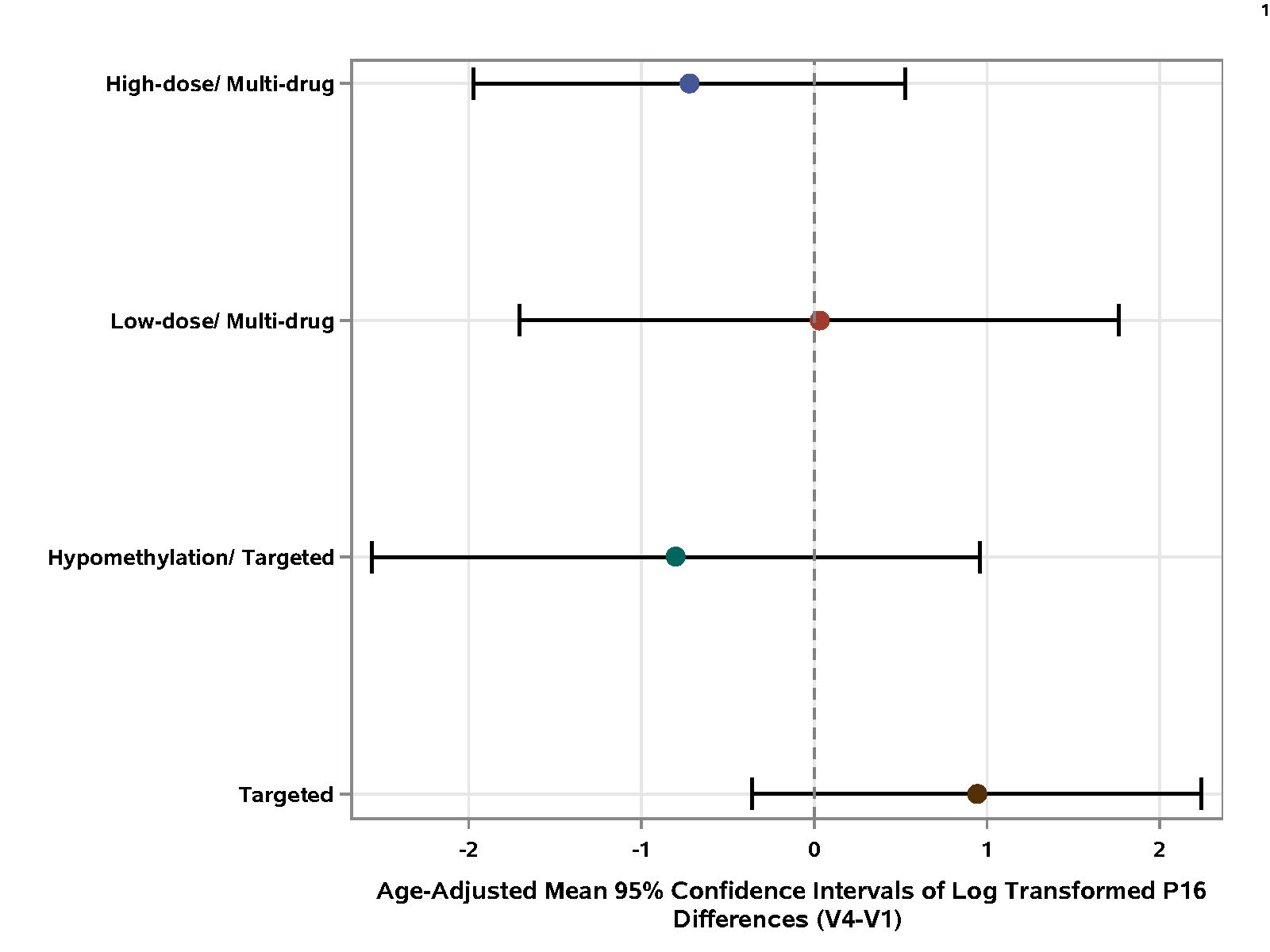

Supplement: Supplementary file 3 — Supplementary file3 (JPG 113 KB) [file 11764_2024_1591_MOESM3_ESM.jpg]

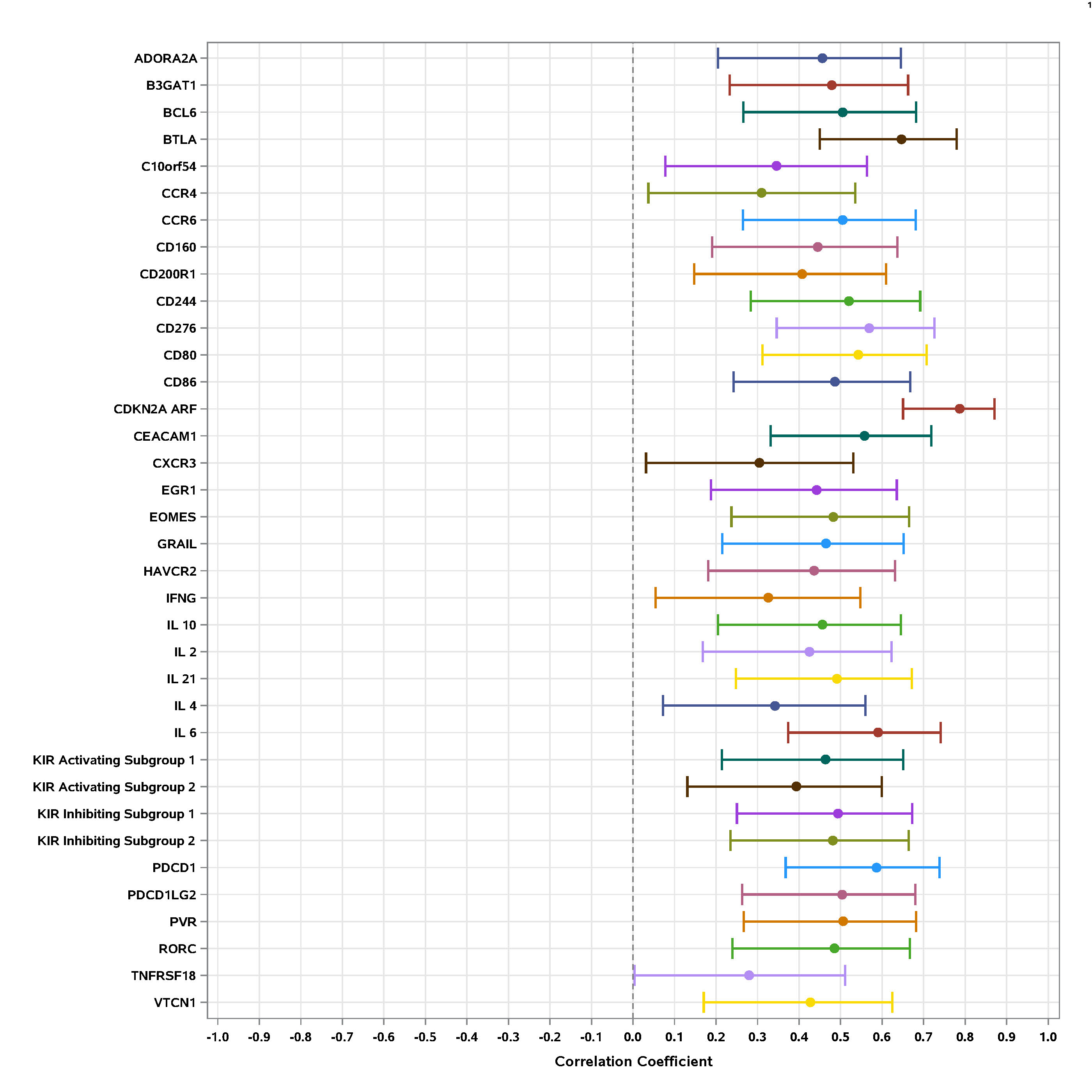

Supplement: Supplementary file 4 — Supplementary file4 (JPG 549 KB) [file 11764_2024_1591_MOESM4_ESM.jpg]
